# Notes and News

**Published:** 1887-05-14

**Authors:** 


### Notes and News.

COLLECTED AND COMMUNICATED.

ON Friday last Lord and Lady Wolverton paid a visit of inspection to the Morley Convalescent Home at St. Margaret's, Dover. The new wing is now in course of erection.

MRS. INGRAM, widow of the founder of the *Illustrated London News*, has contributed a sum of £50 towards the enlargement fund of the Boston Hospital.

MR. J. S. MORGAN has generously made his conuitional donation of £10,000 to Guy's an absolute gift. In the course of a letter he has addressed to the treasurer, he remarks that he only made the stipulation "to stimulate to further exertion in the good work of putting Guy's Hospital into such a state of efficiency as would enable it to meet to the fullest extent the demands of the poor and suffering upon it." Guy's appeal for £100,000 has now been met to the extent of £72,000.

THE Chelsea Hospital for Women is fortunate in possessing many friends, who are always ready to lend the institution a helping hand. The Irving Amateur Dramatic Club, of which Mr. Henry Irving is the president, have kindly promised to give a performance of R. H. Horne's tragedy, "The Death of Marlowe," and a three-act comedy by S. Grundy, entitled "The Silver Shield," in aid of the hospital funds. The performance takes place at St. George's Hall, Langham Place, on Tuesday, 17th inst.

THERE are few people who cannot help the hospitals, if they only will. Here is a pleasing item of news from Yorkshire :—A few young girls of Dewsbury and Batley Carr, hearing that the Heckmondwike Children's Convalescent Infirmary was in need of help, resolved to try what they could do. So they enlisted the sympathies of their parents and friends, and set to work with their needles to make sundry pretty fancy-fixes for a bazaar. In course of time they got together a large number of suitable articles, succeeded in obtaining the free loan of a hall, held their bazaar, and handed to the infirmary the sum of £13. The names of these clever little maidens deserve record; they are Miss Booth, Miss Blackburn, and Miss Lister of Batley Carr; and Miss Bray, Miss Ratcliffe, Miss Dixon, and Miss Fitton of Dewsbury.

WE would remind all who are engaged or interested in hospital work that the annual meeting of The Hospitals Association will be held on Wednesday, the 18th inst., at the Society of Arts, John Street, Adelphi, at five p.m., when Sir Andrew Clark will preside. Ladies and gentlemen interested in hospitals, as well as in the relief of the sick and suffering poor, are cordially invited to attend this meeting.

"A TRAINED NURSE" writes on a blank sheet of paper : "Can anyone kindly give me information concerning the Trained Nurses' Annuity Fund mentioned in THE HOSPITAL?" The envelope bears the postmark Aberfeldy, but as we imagine a letter addressed to "Trained Nurse, Aberfeldy," would be return'd through the dead-letter office, we shall be glad if our correspondent will send her name and address. Once more we must protest, humiliating though it be, that the editorial power does not include the spontaneous divination of the names and addresses of anonymous correspondents.

THE new hospital of the Jaffa Medical Mission was completed and formally opened in October last, greatly to the relief and joy of the ladies in charge, who have suffered much anxiety from frequent interruptions to the work. A report of the mission from August 1885 to December 1886 has been issued, and shows the receipts (including a loan of £255) to have been £1,209, whilst the expenditure was £1,140, thus leaving an apparently favourable balance of £69. The total number of attendances by patients during the period from November 1st 1885 to December 31st 1886 was 11,176; and of 231 patients nursed in the hospital, 12 died, of whom 7 were admitted in a hopeless condition. Of the in-patients there were 183 Moslems, 19 Greeks, 10 Maronites, 8 Jews, 6 Protestants, 3 Latins, 1 American, and 1 Copt —figures which conclusively prove the non-sectarian character of the institution.

WHEN Lord Clarendon was Viceroy of Ireland, he observed, during a visit of inspection to Sir Patrick Dun's Hospital, that while to him a hospital filled with the sick was a melancholy object, a hospital with all needful appliances, but with its doors closed against the suffering poor, was a still sadder sight. By bitter irony of fate, this old institution is threatened with this very calamity. It is an endowed hospital, but at the present hour this circumstance acts rather as a hindrance than a blessing. Once the endowment yielded £1,400 a year, but in 1886 only £400 came from investments. The Provost of Trinity College recently explained to a special meeting called to re-awaken public interest that the distress arises from the non-payment of rents,—"a thing not uncommon or unfashionable at the present day." Sickness or suffering is the only qualification for admission to Sir Patrick Dun's Hospital. The claims of this institution do not, however, rest solely upon the fact that it freely heals the sick poor of Ireland: as a great educational establishment it is of the greatest possible advantage to the coming generation of medical men, and therefore to the whole community.

THE following vacancies are announced this week, the amount of the salary being placed in each case after the name of the institution :—

**Resident Medical Officers.**

Blackburn Infirmary. House Surgeon. Doubly-qualified.—£100.
Chichester Infirmary. House Surgeon and Assistant Secretary.—£100.
Lincoln County Asylum. Medical Superintendent. Duly registered.—£400.
Liverpool Stanley Hospital. Senior House Surgeon. Doubly-qualified.—£80.
Rochdale Infirmary. One. Doubly-qualified.—£80.
Stafford Infirmary. Assistant House Surgeon.—No salary.
Teignmouth Infirmary. House Surgeon and Dispenser. Doubly-qualified.—£71.
Ventnor Consumption Hospital. Dispenser as Locum Tenens.

**Non-resident Medical Officers.**

Charing Cross Hospital. Assistant Surgeon. F.R.C.S.E.
Do. do. Medical Register. Qualified.
Hampstead Consumption Hospital. Two Clinical Clerks.
National Dental Hospital. Assistant Dental Surgeon. L.D.S.
St. Thomas's Hospital Medical School. Professor of Systematic Physiology.
St. Mary's Hospital. Physician for out-patients.

**Matrons, etc.**

Addenbrooke's Hospital, Cambridge. Matron.—£70.
Royal Hospital for Children and Women. Sister Superintendent.—£50.

**Trained Nurses.**

Bradford Eye and Ear Hospital. One.—£25.
Hertford Infirmary. Assistant.—£16 to £20.
Huddersfield Birkby Fever Hospital. Two.—£26 and £20 16s.
Lowestoft Hospital. One Night.—£25.

**Probationer.**

Hertford Infirmary. One.—£12.

---

THE last sessional meeting at The Hospitals Association is looked forward to with much interest by members of the medical profession as well as by hospital managers. Sir Andrew Clark will preside, and Dr. Collier of Oxford will read an interesting and exhaustive paper on Hospital Charity, in which he describes the nature of hospital abuse, the evils to which it gives rise, and the best means of preventing it. The meeting will be held at the rooms of the Medical Society, Chandos Street, Cavendish Square, near the Langham Hotel, and a very large attendance is expected. The paper is moderate in tone, exhaustive in scope, and probably the most weighty utterance which has yet been made on this important subject.

---

THE annual meeting of the Hospital for Epilepsy and Paralysis, Regent's Park, was held last week at the Eyre Arms Assembly Rooms. The report, read by the secretary, Mr. Howgrave Graham, the adoption of which was moved from the chair by General Maclagan, R.E., laid stress on the great need of funds generally prevailing among the London hospitals, and congratulated the supporters that the debt had only increased by £60 during the past year. He expressed the hope that the "Jubilee" year, which marked among the Jews a time for "setting free from bondage," would be characterised by a similar liberation of the hospitals from the "bondage of debt." The Rt. Rev. Bishop Perry seconded the motion. The re-appointment of the retiring members of committee was moved by Sir. W. Magnay, Bart., who said that the administration by his colleagues of the funds at their disposal was a far easier task than the collection of funds to administer. Mr. R. B. Chapman alluded, as a regular visitor to the wards, to the distressing nature of the diseases treated in the hospital, and to the great need of such an institution.

---

AN instance of long-borne gratitude is furnished by the will of the late Mr. Robert Cocks, of Wilby House, Notting Hill. In the year 1832 the deceased gentleman was injured through the overturning of a stagecoach near Gloucester. He was taken to a house in the town, and there for seven weeks he was tended by the late Mr. William Cother, then surgeon to the Gloucester Hospital. In remembrance of this incident, and as a token of his gratitude for the great kindness and attention he then received, Mr. Cocks has left £200 to this hospital.

---

THE National Pension Fund is steadily growing, and many lady superintendents are sending in the names of their staff of nurses, a step also taken by many individual nurses in various parts of the country. The nursing institutions are also joining the movement, and thirty-five nurses of the General Nursing Institute, 4, Henrietta Street, Covent Garden, have sent in a signed statement, to the effect that they desire to express their cordial approbation of the proposed scheme for the foundation of an Annuity Fund, and pledge themselves to support it according to their various means. There are now quite two hundred nursing institutions in England and Scotland, and we should like to receive a similar memorial from the hundred and ninety-nine. If they take this course the Annuity Fund will become a fact, and not a possibility merely, as at present. We propose to publish the names of the institutions, and of the officials who have intimated their willingness to join the Fund, in the number for June 4th next, and any of the institutions wishing to appear in the list should communicate with Mr. Collins, the Secretary of The Hospitals Association, on or before Tuesday, the 31st inst.

---

WE would remind our readers that the Annuity Fund is for officials of all grades as well as for nurses and lady superintendents,—a point which seems to be overlooked, if we may judge from the silence of the secretaries and other officials to this date. Anyone who devotes the best years of his or her life to the cause of suffering humanity, in whatever capacity, and renders faithful service, should be entitled to receive adequate provision for declining years and the days of ill-health or infirmity. This being so, it has been decided to endeavour to make the Annuity Fund a National Fund for all engaged in this good work ; but the silence of the secretaries and other officials leads to the belief that they are indifferent to the movement, and consider themselves not to need any such provision or assistance, despite the excellent reasons to the contrary given by Mr. Newton H. Nixon and Mr. P. Michelli, the Secretaries of University College and St. Mary's Hospitals respectively. If the metropolitan secretaries do not desire to participate in a Pension Fund, are the provincial secretaries of the same opinion ? We cannot credit it, and shall hope to receive the names of many before the end of the present month.

The following sums have been recently received by the various Medical Charities, the name of the donor or the source from which they are obtained being given in each instance after the name of the Institution :—

**Donations.**

Brompton Hospital for Consumption. Mrs. Harris, £10 10*s*. Mrs. Joicey, £10.
Dental Hospital of London, Leicester Square. Mr. F. H. Dutton, £10 10*s*.
Great Northern Central Hospital, Caledonian Road. Vestry of All Hallows, £25. Mr. C. Washington Eves, £31 10*s*.
Hospital for Epilepsy and Paralysis, Regent's Park. The Vestry of St. Martin's Ongar, £5 5*s*.
London Hospital. The Skinners' Company, £21.
North London Consumption Hospital, Hampstead. Mr. A. Hoare, £10 10*s*.
North London Hospital. Miss Flora Goldsmid, £36 15*s*. Miss Isabel Goldsmid, £30. Miss Emma Goldsmid, £27 6*s*. Messrs. Whitbread & Co., £26 5*s*. Mr. Richard Worsley, £20. Mr. W. F. Low, £10 10*s*.
Seamen's Hospital, Greenwich. Governors of the London Assurance Co., £10 10*s*. Mr. Cosmo Romilly, £10 10*s*. Mr. John Bramwell, £10 10*s*. Hon. A. Tollemache, £100. Leathersellers' Company, £21. Society of Apothecaries, £21. Alderman Stuart Knill, £10 10*s*. Messrs. Frederick Huth & Co., £10 10*s*.
The Mary Wardell Convalescent Home for Scarlet Fever. Mr. John Proctor, £10 10*s*.

**Legacies.**

Beccles Hospital. Mr. Robert Cocks, £100.
Brixton Hospital. Mr. Robert Cocks, £100.
London Hospital. Mr. Robert Cocks, £100.
Norwich and Norfolk Hospital. Mr. Robert Cocks, £100.
Royal Free Hospital. Mr. Robert Cocks, £100.
Royal Hospital for Incurables, Putney. Mr. Robert Cocks, £100.
The Gloucester Hospital. Mr. Robert Cocks, £200.

---

In the parish of St. Margaret, Westminster, the Queen's Jubilee is to be commemorated in two forms—(1) the erection of a porch at the parish church, and (2) the endowment of beds for sick children at Westminster Hospital. It is significant of the drift of public sympathy when we say that, while the subscriptions to the first project only amount to about £250, the subscriptions to the latter amount to about £1,500. We learn that the Committee of Westminster Hospital have arranged to affix on the outside of the building a tablet recording the memorial thus provided for.

---

The second annual report of the Kensington District Nursing Association bears very strong evidence of the increasing good work which this little institution is accomplishing among a huge population. During the first year of its existence 168 cases were attended by the nurses, and 3,202 visits paid, while last year there were 418 patients attended, involving about 9,000 visits. The timely assistance rendered by the nurses of this Association to the sick poor frequently saves them from being compelled to enter the hospital or infirmary, so that the institution may be regarded in the light of a valuable auxiliary to our medical charities. The poor are, through their complete ignorance of the elements of nursing, very helpless in time of sickness. The presence of a skilled nurse at such a moment therefore serves a double blessing, for it not only saves the sick man from much suffering, but the hospital probably from the cost of an in-patient. The Kensington District Nursing Association, of which Miss Louisa Twining is one of the hon. secs., is capable of very extensive development, and as the splendid work of its devoted nurses becomes more widely known, we do not doubt that the public will accord it increasing support.

The West London Hospital still labours under a heavy incubus of debt, amounting now to £5,800. Mrs. Hull Martin, who annually sends a cheque for £100 to this hospital, has just come forward with an offer of £100 towards a fund for extinguishing the debt remaining over from last year. Another friend, Mr. C. S. Young, offers £50 for the same purpose if five others will each give a similar amount. Special departments have recently been opened at the West London for the diseases of women, the eye, teeth, ear, throat, skin, and orthopædic cases.

---

The recent annual meeting of the supporters of the Felixstowe Convalescent Home revealed a happier state of things than is the case at many such institutions. The new wing, which cost £3,650, has been paid for, and the Home is now wholly clear of debt, and this, too, despite the deaths and removals of several old friends and subscribers. Last year 398 patients were admitted to the Home, all of whom received great benefit from their stay. We are glad to see that it has been decided to open the Home in future from the 1st April till the 1st January, instead of from 1st May to 1st December as hitherto. Suffolk folk are very thrifty, and their example in this case is worthy of general imitation.

---

The luxury of doing good in one's lifetime, when it involves the expenditure of a substantial portion of our wealth, is one in which but few men indulge. We give our stereotyped guinea or five guineas annually to some hospital; our conscience approves itself to us, and so the years roll on till the sands of the hour-glass have almost run their course. Then we turn over in our minds the question of the disposition of our savings, and, after remembering our friends and servants, allot our money to this, that, and the other hospital. Far be it from us to say a word against those who make posthumous benefactions, for to the sick poor they are the same; but those who choose this mode of doing good to our fellow-men lose one of the grandest pleasures we can conceive. Mr. John Fleming, of Newcastle, has reserved to himself the luxury of doing good in his own lifetime. The old Tyneside Hospital for Children in Hanover Square, Newcastle, has long been insufficient for the growing requirements of this town. Twenty-four beds, which were all that the old institution possessed, were totally inadequate to meet the increasing requirements of the town. At this juncture Mr. Fleming stepped forward, and, in memory of his late wife, generously volunteered to give Newcastle a Children's Hospital. Including the cost of the site, the building, and the furnishing, the necessary expenditure will probably reach upwards of £20,000. Lady Armstrong, whose sympathy with the sick little folk of Newcastle is well known, most suitably performed the interesting ceremony of laying the foundation stone of the Fleming Memorial Hospital, as the new institution at Moor Edge is to be called. The hospital, which is being erected by Messrs. J. and W. Lowry, will prove a great architectural adornment to the town. The new building will provide sixty beds, and also the advantages of a convalescent home.

